# Creation of Cross-Linked Crystals With Intermolecular Disulfide Bonds Connecting Symmetry-Related Molecules Allows Retention of Tertiary Structure in Different Solvent Conditions

**DOI:** 10.3389/fmolb.2022.908394

**Published:** 2022-06-08

**Authors:** Takeshi Hiromoto, Teikichi Ikura, Eijiro Honjo, Michael Blaber, Ryota Kuroki, Taro Tamada

**Affiliations:** ^1^ Institute for Quantum Life Science, National Institutes for Quantum Science and Technology, Ibaraki, Japan; ^2^ Institute for Human Life Science, Ochanomizu University, Tokyo, Japan; ^3^ Research and Development Division, ADTEC Co., Ltd., Oita, Japan; ^4^ College of Medicine, Florida State University, Tallahassee, FL, United states; ^5^ Quantum Beam Science Center, Japan Atomic Energy Agency, Ibaraki, Japan

**Keywords:** T4 phage lysozyme, crystal immobilization, disulfide cross-linking, x-ray crystallography, pH tolerance, osmotic tolerance

## Abstract

Protein crystals are generally fragile and sensitive to subtle changes such as pH, ionic strength, and/or temperature in their crystallization mother liquor. Here, using T4 phage lysozyme as a model protein, the three-dimensional rigidification of protein crystals was conducted by introducing disulfide cross-links between neighboring molecules in the crystal. The effect of cross-linking on the stability of the crystals was evaluated by microscopic observation and X-ray diffraction. When soaking the obtained cross-linked crystals into a precipitant-free solution, the crystals held their shape without dissolution and diffracted to approximately 1.1 Å resolution, comparable to that of the non-cross-linked crystals. Such cross-linked crystals maintained their diffraction even when immersed in other solutions with pH values from 4 to 10, indicating that the disulfide cross-linking made the packing contacts enforced and resulted in some mechanical strength in response to changes in the preservation conditions. Furthermore, the cross-linked crystals gained stability to permit soaking into solutions containing high concentrations of organic solvents. The results suggest the possibility of obtaining protein crystals for effective drug screening by introducing appropriate cross-linked disulfide bonds.

## Introduction

In general, protein crystals are mechanically fragile and sensitive to subtle changes such as pH, ionic strength, and/or temperature in their crystallization mother liquor. One of the major reasons is that they contain a substantial number of cavities and channels occupied by solvent molecules that participate in intermolecular crystal contacts. It is assumed that 20−80% of the crystal volume is made by solvent molecules (Matthews, 1968; Carugo et al., 2017). Whereas the high solvent content of protein crystals, leading to their mechanical weakness, may cause difficulties in handling them and in using them in applications as a material, it allows the diffusion of small substances into the crystals by soaking techniques. Such crystal soaking is widely performed in protein X-ray crystallography to prepare substrate-enzyme complexes and to search potential lead compounds for drug discovery.

When introducing substrates into enzyme crystals *via* soaking, the catalytic reactions can proceed even in the crystalline state (Quiocho et al., 1972; Acheson et al., 2017). However, the enzymes are not always crystallized under their optimal pH values. Sometimes it is desirable to change the pH value of the preservation solution, but crystal soaking into a solution different from that for growing crystals can cause severe damage to the crystals, leading to loss of their diffraction ability. In order to obtain the crystal structure of the complex of protein and lead compound, the protein crystals must be soaked into a solution containing an organic solvent because of the hydrophobic properties of many lead candidates. Of course, a high concentration of organic solvent such as alcohol or dimethyl sulfoxide (DMSO) dissolves protein crystals by weakening the packing interactions between neighboring molecules (Sugiyama et al., 2012). Therefore, it can be advantageous to produce rigid protein crystals not affected by the soaking operation into different solution conditions. Several approaches have been reported so far to improve the mechanical stability of protein crystals, including growing in a high-concentration hydrogel (Sugiyama et al., 2012; Maruyama et al., 2016), chemical cross-linking (Yan et al., 2015, 2016; Kubiak et al., 2019; Nguyen et al., 2019), and rational design of crystal-packing contacts (Heinz and Matthews, 1994; Derewenda, 2004; Yamada et al., 2007; Abe and Ueno, 2015; Negishi et al., 2018; Hermann et al., 2021). Considering that chemical cross-linkers sometimes cause non-specific reactions with essential residues for enzymatic activities (Ayala et al., 2002), rational design such as the introduction of disulfide bridges based on the structural information could be more suitable in immobilizing protein crystals while preserving their activities. Typically, protein molecules are densely and regularly arranged in a crystal lattice by using intermolecular interactions which are much weaker than the intramolecular interactions (Carugo et al., 2017). It can be expected that the rational design of the packing contacts by introducing covalent disulfide bonds would enforce assembly of proteins in the crystals, leading to reduced damage to the crystals caused by soaking into different preservation conditions.

In general, disulfide cross-links engineered into proteins do not always enhance their stability, because unfavorable contacts can be occurred in the surrounding residues around the disulfide bond or existing favorable interactions can be lost within the folded protein (Dani et al., 2003; Hibi et al., 2016). The prediction of appropriate positions for disulfide cross-linking is still difficult. Meanwhile, there are successful examples of stabilizing a protein by introducing internal-disulfide bonds (Matsumura and Matthews, 1991; Siadat et al., 2006; Lee and Blaber, 2009). Introducing external-disulfide bonds into a protein has also been conducted to produce a dimeric structure or to promote fiber-like assembly (Heinz and Matthews, 1994; Yang et al., 2000; Banatao et al., 2006). Using T4 phage lysozyme (T4L) as a model protein, we attempt here to immobilize it three-dimensionally in its crystal lattice by disulfide cross-linking and to evaluate the effect of cross-linking on the stability of the crystals by microscopic observation and X-ray diffraction. T4L is one of the well-studied enzymes in its function (Kuroki et al., 1993, 1995, 1999; Hiromoto et al., 2017) and stability (Rennell et al., 1991; Shoichet et al., 1995; Baase et al., 2010) interpreted in terms of the tertiary structure. Many mutation studies have been carried out based on the cysteine-free pseudo-wild type T4L (TA), carrying the C54T and C97A mutations (Matsumura et al., 1989), to characterize the functions of selected residues.

In this study, four residues (Ser44, Asn68, Ala93, and Thr115) located at the crystal packing contacts of TA were mutated to cysteine (designated as SNAT-T4L) so that all neighboring molecules in the crystal can be bridged by intermolecular disulfide cross-links. The obtained cross-linked crystals had a diffraction resolution of approximately 1.1 Å resolution, comparable to that of the non-treated crystals. Furthermore, they maintained their diffraction ability even when immersed into precipitant-free solutions ranging across several pH values, or with high concentrations of organic solvents.

## Materials and Methods

### Protein Expression and Purification

The SNAT-T4L gene carrying four point mutations (S44C, N68C, A93C, and T115C) was prepared by manipulating the TA gene background (Matsumura et al., 1989). After subcloning the DNA fragment into a pHN1403 ampicillin-resistant plasmid (Poteete et al., 1991), *Escherichia coli* strain RR1 was transformed with the construct containing the SNAT-T4L gene, and then the positive transformants were screened for protein expression. The cells harboring the plasmid were cultivated in LB medium at 37°C until reaching an optical density of 0.6 at 600 nm and protein expression was induced for an additional 1.5 h at 30°C by adding isopropyl-β-D-thiogalactopyranoside (IPTG) to the culture medium at a final concentration of 1 mM. The cells were harvested by centrifugation at 8000 g for 15 min at 4°C and then were suspended in 50 mM Tris-HCl buffer (pH7.5) containing 1 mM dithiothreitol (DTT) and were disrupted by sonication at 200 W for 20 min using a Kubota Insonator Model 201M (Kubota Co., Japan). After centrifugation of the cell lysate, the supernatant was applied to a CM-Sepharose FF column (GE Healthcare), and the eluate containing SNAT-T4L was further purified by a Resource S column (GE Healthcare), where the absorbed proteins were eluted from the column by a linear gradient of 0.05−0.35 M NaCl in 50 mM Tris-HCl buffer (pH7.2) containing 1 mM ethylenediaminetetraacetic acid (EDTA) and 1 mM DTT.

### Crystallization

The SNAT-T4L crystals were obtained by hanging drop vapor diffusion against a reservoir solution composed of 0.1 M Tris-HCl (pH6.8), 2 M Na-K phosphate, 0.1 M NaCl, 0.35% (*v*/*v*) 1,6-hexanediol, and 15 mM DTT as described previously (Kuroki et al., 1993, 1995). A mixture of 20 μL of the purified enzyme (9 mg/mL) and 20 μl of the reservoir solution was equilibrated at 20°C for two weeks. Once crystals were formed, copper sulfate as an oxidizing agent was added to the crystallization mother liquor to be a final concentration of 0.01% (*w*/*v*). These crystals were then left to stand for 20°C for a week to achieve disulfide cross-links between molecules in the whole crystal. To confirm the mechanical strength of the cross-linked crystals, they were soaked into distilled water, precipitant-free solutions with different pH values (pH2−12), and precipitant-free solutions containing several organic solvents. The pH effects on the cross-linked crystals were investigated by soaking them into the precipitant-free solutions, which were prepared with 50 mM each of the following buffers: citrate at pH2−5; phosphate at pH6 and 7; tricine at pH8; borate at pH9−12, in addition to 0.2 M NaCl and 30% (*v*/*v*) glycerol but not precipitant. To investigate osmotic effects, the cross-linked crystals were soaked for 12 hr into the precipitant-free solutions containing different concentrations of dimethyl sulfoxide (DMSO; 0−50% *v*/*v*), 50 mM phosphate buffer at pH7, 0.2 M NaCl, and 30% (*v*/*v*) glycerol. In only the case using 30% DMSO, back-soaking the crystals in a precipitant-free solution without DMSO was required to recover their diffraction ability. Such soaking experiments using ethanol and acetonitrile, commonly used for protein purification, were also conducted.

### Diffraction Data Collection, Processing, and Structure Determination

Crystals were immersed in a cryoprotectant solution supplemented with 30% (*v*/*v*) glycerol prior to X-ray diffraction experiments. The diffraction data were collected at the beamlines NW12A at Photon Factory Advanced Ring (PF-AR) (Tsukuba, Japan), BL5A at Photon Factory (PF), and BL41XU at SPring-8 (Harima, Japan) under a cold nitrogen gas stream (100 K). Each diffraction data set was indexed, integrated, and scaled using the *HKL2000* software package (Otwinowski and Minor, 1997). The statistics for data collection are summarized in Tables 1 and S1. Crystal structure refinement was performed using the TA structure (PDB ID: 5VNR) as an initial coordinate with *phenix.refine*, implemented in the *PHENIX* software package (Adams et al., 2010). Individual anisotropic atomic displacement parameters were refined for diffraction data, excluding 20% DMSO data. After several rounds of iterative manual rebuilding of a protein model, water picking was conducted based on the 2*F*_o_ − *F*_c_ and *F*_o_ − *F*_c_ electron-density maps using *Coot* (Emsley et al., 2010). The refinement statistics of each final model are also summarized in Table 1.

**TABLE 1:** Data collection and refinement statistics for the SNAT-T4L mutant in different buffer conditions. The statistics for the highest-resolution shell are presented in parentheses.

	pH4	pH7	pH10	DMSO 20%	DMSO 40% (Back-Soaked)
Data collection					
Beamline	NW12A at PF-AR	NW12A at PF-AR	NW12A at PF-AR	BL5A at PF	BL5A at PF
Cell constants (Å)	*a* = *b* = 60.30, *c* = 96.87	*a* = *b* = 60.00, *c* = 97.10	*a* = *b* = 59.98, *c* = 97.07	*a* = *b* = 59.87, *c* = 96.03	*a* = *b* = 59.90, *c* = 96.77
Resolution (Å)	32.3 − 1.30 (1.35 − 1.30)	45.9 − 1.10 (1.14 − 1.10)	26.0 − 1.05 (1.09 − 1.05)	25.9 − 1.50 (1.55 − 1.50)	35.4 − 1.10 (1.14 − 1.10)
Unique reflections	50560 (4785)	82294 (8082)	93665 (8688)	32395 (3067)	81025 (7157)
Redundancy	10.1 (6.6)	9.6 (9.2)	10.0 (6.4)	10.3 (7.7)	9.8 (5.5)
*I*/σ (*I*)	26.0 (2.6)	21.0 (2.5)	27.0 (3.1)	30.7 (3.8)	23.3 (2.1)
*R*_meas_ ^a^	0.081 (0.591)	0.066 (0.609)	0.054 (0.401)	0.054 (0.465)	0.062 (0.630)
Completeness (%)	99.6 (95.9)	99.5 (99.0)	98.8 (92.5)	99.5 (95.8)	98.7 (88.4)
*CC*_1/2_ ^b^	0.998 (0.840)	0.999 (0.891)	0.999 (0.925)	0.999 (0.949)	0.999 (0.812)
Wilson *B* (Å^2^)	13.16	9.97	10.96	21.39	11.94
Refinement					
Resolution (Å)	32.3 − 1.30 (1.33 − 1.30)	45.9 − 1.10 (1.13 − 1.10)	26.0 − 1.05 (1.08 − 1.05)	25.9 − 1.50 (1.54 − 1.50)	35.4 − 1.10 (1.13 − 1.10)
Used reflections	50487 (3366)	82190 (5761)	93563 (6016)	32290 (2165)	80732 (4908)
*R*_work_ ^c^	0.153 (0.231)	0.148 (0.182)	0.164 (0.193)	0.202 (0.252)	0.159 (0.235)
*R*_free_ ^d^	0.171 (0.258)	0.168 (0.183)	0.178 (0.221)	0.232 (0.306)	0.181 (0.239)
RMSD					
bond length (Å)	0.006	0.006	0.005	0.008	0.006
Bond angle (°)	0.977	1.001	0.968	1.216	0.953
No. of atoms	1654	1731	1677	1552	1707
Macromolecules	1369	1383	1363	1352	1413
Waters	245	317	294	148	270
Others	40	31	20	52	24
Mean *B* value (Å^2^)	19.70	16.06	17.77	27.40	18.84
Macromolecules	16.80	12.86	14.96	25.89	16.24
Waters	31.91	28.45	29.18	38.97	31.87
Others	44.20	32.45	41.75	33.63	25.45
PDB code	7XE5	7XE6	7XE7	7XE9	7XEA

a R_meas_ is the multiplicity-weighted R_merge_. R_merge_ = *Σ* | I_hkl_ - <I_hkl_> | / *Σ* I_hkl_, <I_hkl_> is the mean value of I_hkl_.

b CC_1/2_ values were calculated by splitting the data randomly in half.

c R_work_ = *Σ*| | F_o_ | − | F_c_ | | / *Σ* | F_o_.

d R_free_ was calculated from the test set (5% of the total data).

## Results and Discussion

### Rational Design of Intermolecular Disulfide Bonds in Protein Crystals

**FIGURE 1:**
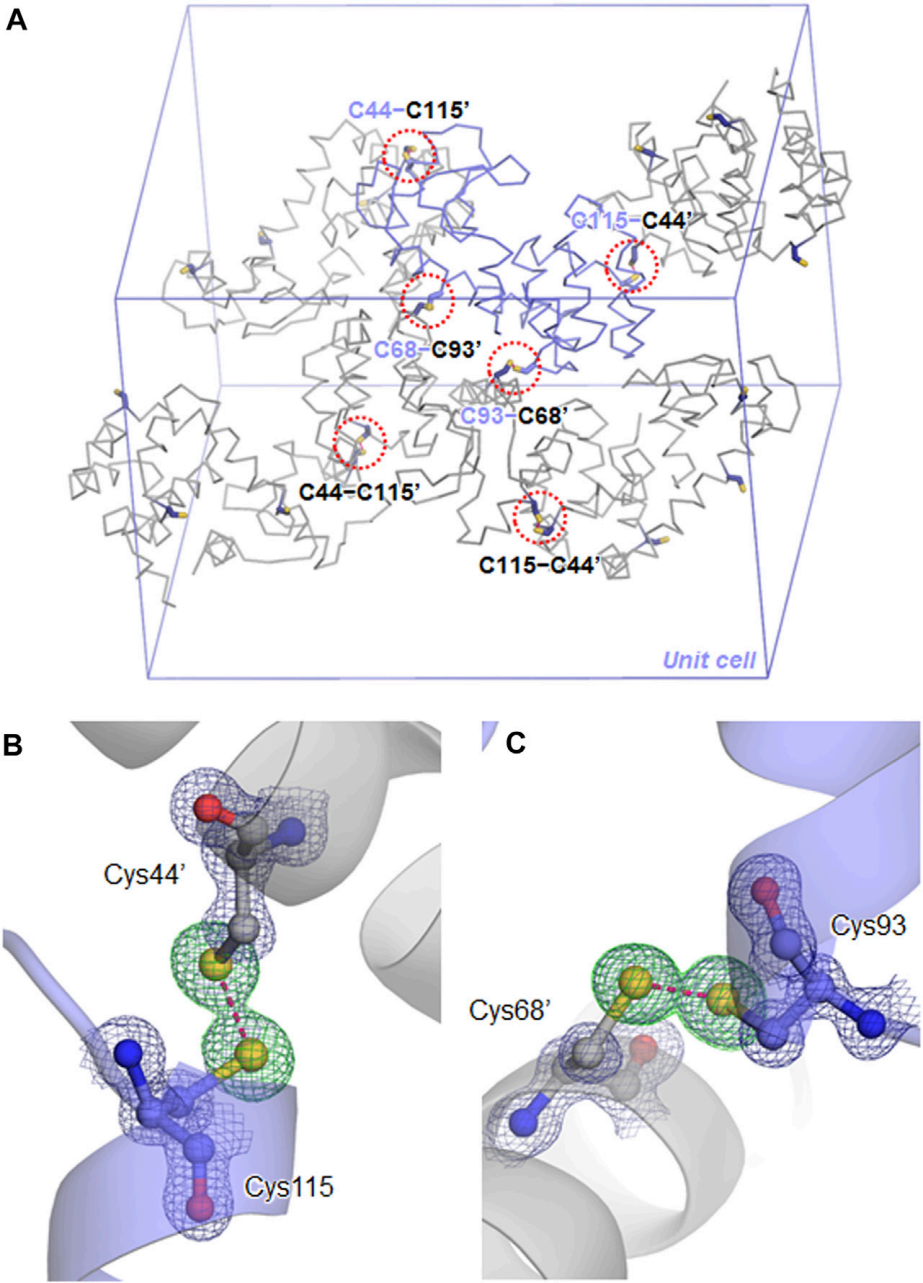
Disulfide cross-links in the SNAT-T4L crystal. **(A)** Symmetry-related molecules in the *P*3_2_21 unit cell are shown in backbone representations. One independent molecule is colored in slate, and the others are in gray. Residues mutated to cysteines at the packing contacts are shown by sticks, where the prime symbol denotes a residue from the symmetry-related molecule. Each red dashed circle indicates the locations of disulfide cross-links in the unit cell. **(B)** The C115−C44’ disulfide bond, superimposed with a 2*F*_o_ − *F*_c_ electron-density map contoured at 1.5σ (deep blue mesh). The green mesh shows a *F*_o_ − *F*_c_ omit electron-density map contoured at 4.0σ, calculated for visualizing the sulfur atom positions. The disulfide bond is depicted by a pink dashed line, with a distance of 2.05 Å. **(C)** The C93−C68’ disulfide bond with a distance of 2.03 Å.

Using the cysteine-free pseudo-wild type T4L (TA) as a model protein (Matsumura et al., 1989), the three-dimensional immobilization of protein crystals was conducted by introducing disulfide cross-links between neighboring molecules in the crystal. Previously, the surface residues Asn68/Ala93’ were replaced by cysteines to promote a dimerization that occurs in the crystallization process, where the prime symbol denotes a residue from the symmetry-related molecule (Heinz and Matthews, 1994). The crystal structure of the mutant remained very similar to TA despite the formation of two intermolecular disulfide bridges. In order to connect all the neighboring molecules in the crystal, the other residue pairs were searched based on the distance between the Cβ atoms. Two residue pairs, Thr21/Lys124’ and Ser44/Thr115’, were found as possible candidates with distances shorter than 4.6 Å between the respective Cβ atoms, but the Ser44/Thr115’ pair located on a larger contact area was selected for cysteine mutation (Figure 1A). This mutant, designated as SNAT-T4L, was purified as a monomer protein under reducing conditions and then was crystallized in the same space group under similar crystallization conditions at pH6.8 to that of TA. The disulfide cross-linking was subsequently achieved by soaking copper sulfate as an oxidizing agent directly into the crystal.

### Crystal Structure of SNAT-T4L in the Cross-Linked Crystals

**FIGURE 2:**
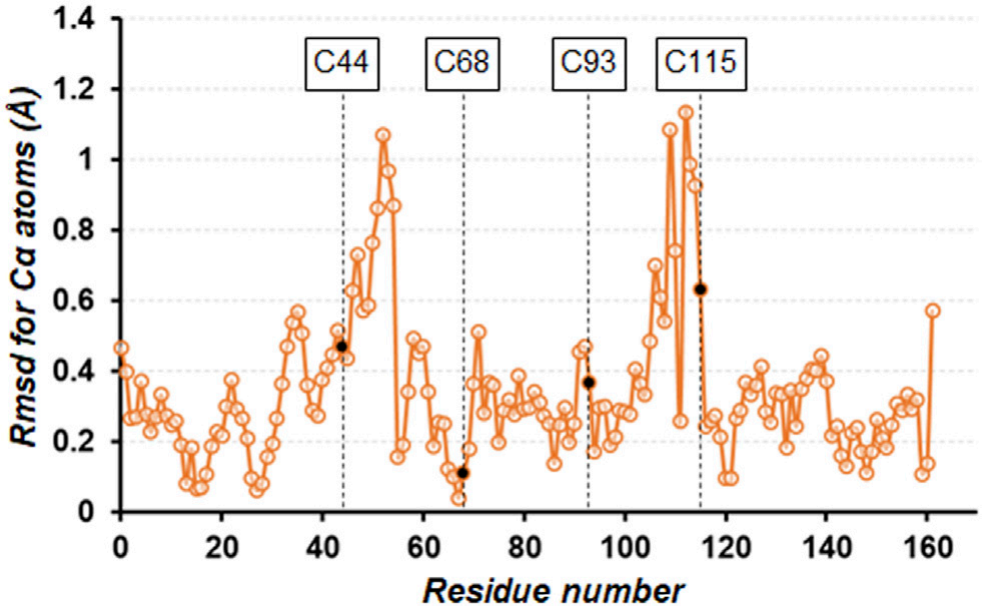
Plot of the rmsd values for Cα atoms of the cross-linked SNAT-T4L structure with respect to that of TA (PDB ID: 5VNR). The four mutation sites are indicated by black circles.

After immersing the cross-linked crystals into a precipitant-free solution containing 50 mM phosphate buffer at pH7, 0.2 M NaCl, and 30% (*v*/*v*) glycerol, the X-ray diffraction data were collected under a cold nitrogen gas stream at 100 K. The diffraction patterns were indexed in the space group *P*3_2_21 with cell constants of *a* = *b* = 60.0 Å and *c* = 97.1 Å, where one of the six independent molecules is contained within the asymmetric unit. The unit cell volume was slightly smaller than those of non-treated crystals of TA (*a* = *b* = 61.5 Å and *c* = 95.9 Å) (Li et al., 2017) and another TA-T26H mutant (*a* = *b* = 61.5 Å and *c* = 97.7 Å) (Hiromoto et al., 2017). The structure refinement at 1.10 Å resolution converged to crystallographic *R*_work_/*R*_free_ values of 14.8/16.8% (Table 1). In the unit cell, one independent molecule forms a trimer unit with two neighboring molecules through intermolecular disulfide bonds, Cys44−Cys115’ and Cys115−Cys44’, respectively (Figure 1A). The chemical oxidation time of a week was enough to complete the disulfide formation over all molecules in the crystal. These disulfide bond lengths are estimated as both 2.05 Å based on the electron densities of cysteine sulfur atoms (Figure 1B). One trimer is further connected to another trimer through two disulfide bonds, Cys68−Cys93’ and Cys93−Cys68’, with each bond length of 2.03 Å (Figure 1C). These bond lengths correspond to the typical disulfide bond length. The overall structures of SNAT-T4L and TA (PDB ID: 5VNR) can be superimposed with a root-mean-square deviation (rmsd) of 0.45 Å over 164 aligned Cα positions (Figure 2). Although there are no large conformational differences between the structures, the small displacements of main-chain atoms are seen at the residues behind Cys44 and before Cys115, which could be caused by the formation of intermolecular disulfide bonds, Cys44−Cys115’ and Cys115−Cys44’.

### pH Effects on the Stability of the Cross-Linked Crystals

**FIGURE 3:**
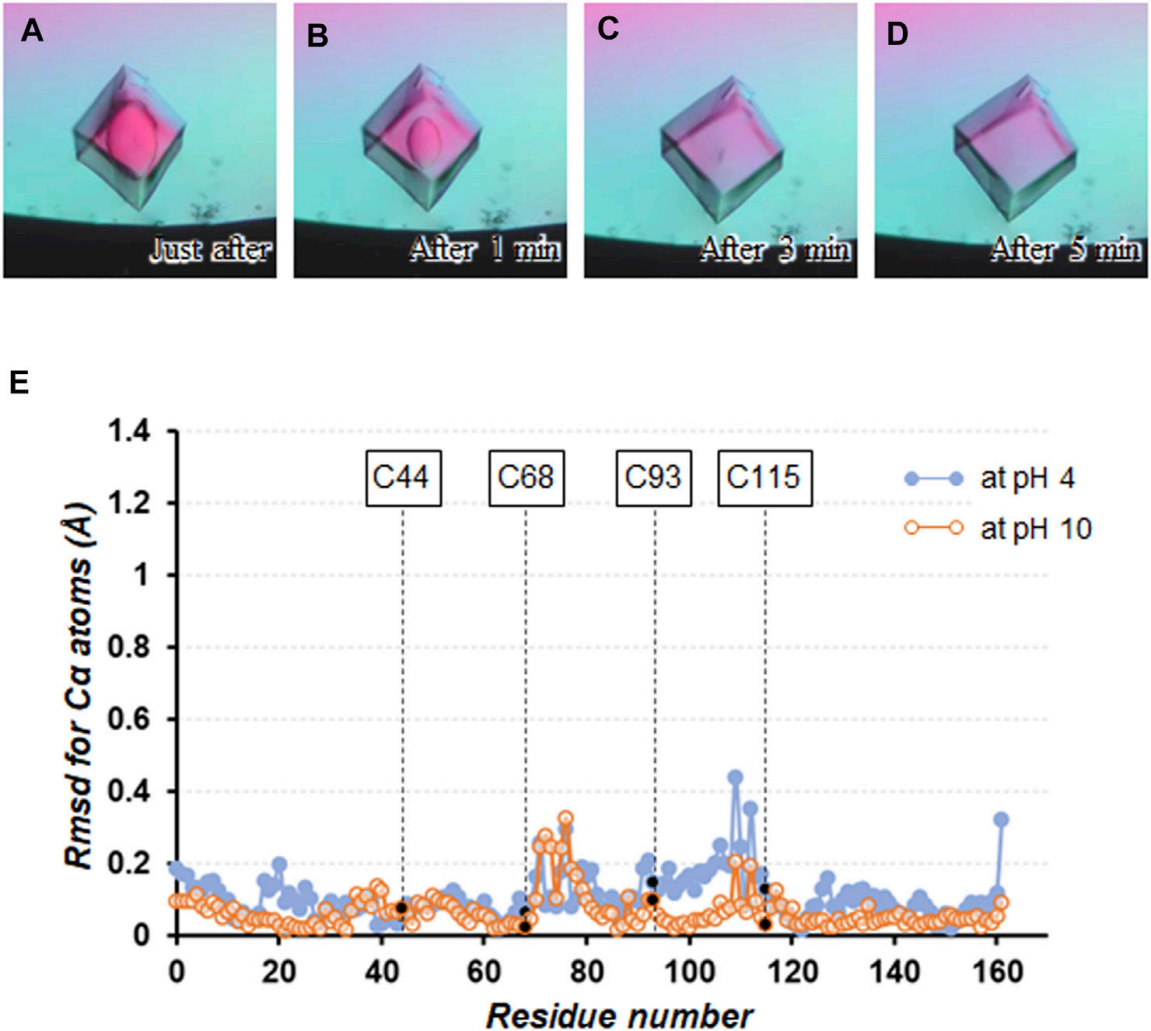
Soaking experiment using a precipitant-free solution with pH3. Crystal images **(A)** just after soaking, **(B)** after 1 min, **(C)** after 3 min, and **(D)** after 5 min are shown. **(E)** Plot of the rmsd values for Cα atoms between the structures at pH4 and 10 with respect to that at pH7.

The pH effects on the stability of the cross-linked crystals were investigated by soaking them into the precipitant-free solutions with different pH values from 2 to 12. When soaking the crystals into the preservation solutions below pH3 and above pH11, the solvent diffusing into the crystal was visualized by microscopic observation as shown in Figures 3A–D, presumably due to the difference of birefringence property between the crystalline and amorphous phases. It took about 5 min to complete the phase change over the entire crystal, of course, depending on the size of the crystals. From these crystals, no X-ray diffractions were observed under cryogenic conditions, indicating their loss of diffraction ability throughout the soaking operation. Because the crystal shapes were maintained during a week of soaking, the acid/base denaturation of proteins in the cross-linked crystals, resulting in the amorphous solid state, could be limited to their partial unfolding (Phan-Xuan et al., 2021). Indeed, pH is known to influence the stability of proteins by altering the net charge on the proteins, and many proteins denature at extremes of pH because of the presence of destabilizing intramolecular electrostatic interactions (Fink et al., 1994; Khurana et al., 1995; Dwyer et al., 2000).

On the other hand, no change in appearance was seen by microscopic observation when soaking the cross-linked crystals into the precipitant-free solutions from pH4 to 10. These crystals diffracted to 1.05 − 1.30 Å resolution (Table 1 and Supplementary Table S1), comparable to the non-cross-linked TA-T26H mutant determined at 1.04 Å resolution (Hiromoto et al., 2017). Comparing the crystal structure at pH7 with that at pH4 and 10, the rmsd values for Cα atoms were calculated as 0.15 and 0.09 Å, respectively, indicating that no significant conformational changes were caused by altering pH (Figure 3E). Each intermolecular disulfide bond was also not affected under all conditions. In a detailed comparison between the structures at pH4 and 7, subtle differences in electrostatic interactions were found in the intramolecular interactions (Table 2). It appeared that most electrostatic interactions were elongated by the lower pH, presumably because the change of the protonation states for Asp (p*K*_a_ = 3.4), Glu (p*K*_a_ = 4.1), and His (p*K*_a_ = 6.5), which can be affected in this pH change (Pahari et al., 2019). Particularly, the loss of electrostatic interactions of N^ζ^(Lys60) − O^ε2^(Glu64) and N^η2^(Arg80) − O^ε1^(Glu108) in the structure at pH4 might be related to the slight displacement of Cα positions between Cys68 and Cys115 as seen in Figure 3E. When the pH becomes more acidic, such weakened interactions (or repulsive forces) may trigger the partial unfolding of protein molecules, leading to the disorder of the crystalline phase. Furthermore, diffraction data were not always obtained from all crystals immersed in the preservation solution with pH4. Because SNAT-T4L has a theoretical pI of 9.43, the effect of lowering to pH4 could be more significant than increasing to pH10.

**TABLE 2:** Comparison of the electrostatic interactions observed in the structures at different pH. Selected interactions within 3.6 Å are listed (Cohen et al., 2008), where their coordinate uncertainties were estimated from the Cruickshank’s DPI (diffraction-component precision index) to be 0.04 Å for the structure at pH4, 0.03 Å for that at pH7, and 0.03 Å for that at pH10, respectively (Vaguine et al., 1999).

Electrostatic interactions	pH4^a^	pH7	pH10^a^
N-term (M1)−O^ε1^ (E64’)	3.15 (+0.06)	3.09	3.06 (−0.03)
O^δ1^ (D10)−N^η2^ (R148)	2.80 (−0.01)	2.81	2.81 (0)
O^ε2^ (E11)−N^η1^ (R145)	2.80 (+0.04)	2.76	2.79 (+0.03)
N^η2^ (R14)−O^ε2^ (E128’)	2.95 (+0.55)	2.40^b^	2.36 (−0.04)
N^ζ^(K19)−O^δ2^ (D127’)	2.91 (+0.12)	2.79	2.79 (0)
O^ε2^ (E22)−N^η1^ (R137)	2.88 (+0.04)	2.84	2.83 (−0.01)
N^δ1^(H31)−O^δ2^ (D70)	2.65 (+0.04)	2.61	2.68 (+0.07)
O^ε1^ (E45)−N^ζ^(K48)	2.89 (+0.08)^b^	2.81^b^	2.89 (+0.08)
O^ε1^ (E45)−N^ζ^(K135’)	2.44^b^	None	None
N^ε^(R52)−O^ε2^ (E62)	2.80 (−0.02)	2.82	2.84 (+0.02)
N^ζ^(K60)−O^ε2^ (E64)	None	2.94^b^	None
O^δ2^ (D72)−N^η1^ (R76)	2.65 (−0.19)^b^	2.84	None
O^δ2^ (D72)−N^η1^ (R96’)	3.36^b^	None	None
N^η2^ (R76)−O^δ1^ (D89)	None	None	2.80
N^η2^ (R80)−O^ε1^ (E108)	None	3.51^b^	2.63 (−0.88)^b^
N^ζ^(K85)−O^δ2^ (D89)	2.79 (+0.10)	2.69	2.63 (−0.06)
O^δ1^ (D92)−N^η1^ (R95)	2.90 (+0.04)	2.86	2.85 (−0.01)
O^δ1^ (D159)−N^ζ^(K162)	None	3.39	None

a Differences between the structures at pH4 and 10 with respect to that at pH7 are in the respective parentheses.

b Either or both residues adopted alternative conformations predumably because the interaction was weakened.

### Osmotic Stresses on the Stability of the Cross-Linked Crystals

**FIGURE 4:**
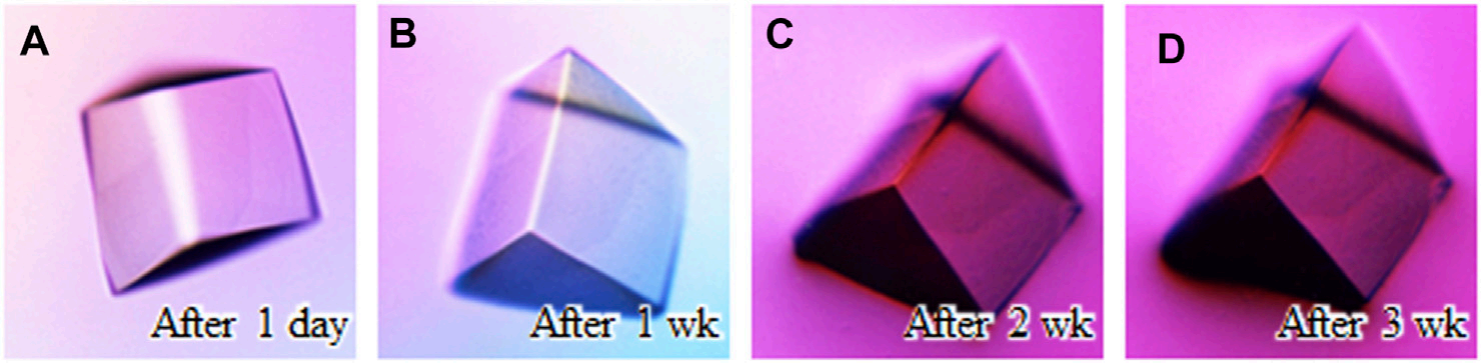
Soaking experiment using a distilled water. Crystal images **(A)** after 1 day soaking, **(B)** after 1 week, **(C)** after 2 weeks, and **(D)** after 3 weeks are shown.

Protein crystals grown by conventional crystallization using precipitants can be fatally damaged by osmotic stress, such as organic solvents, during their soaking operation (Sugiyama et al., 2012). Providing osmotic resistance to protein crystals would be beneficial for drug screening based on protein-lead compound complexes because many lead candidates are insoluble in aqueous solutions. The disulfide cross-linking of protein crystals could be one of the approaches to alleviate this problem. At the beginning, the cross-linked crystals were immersed into a distilled water as a relatively mild change in the osmotic pressure (Figure 4). Despite three weeks of soaking, the crystals maintained their shape and diffracted to 1.05 Å resolution (Supplementary Table S1).

**FIGURE 5:**
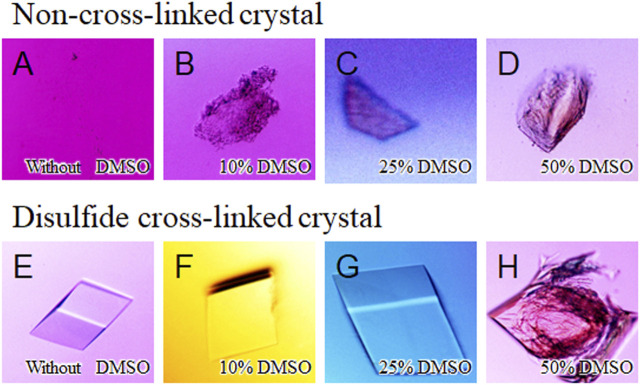
Soaking experiment using different concentrations of DMSO solutions. Images of non-cross-linked crystals immersed in precipitant-free solutions **(A)** with 0% (*v*/*v*) DMSO, **(B)** with 10%, **(C)** with 25%, and **(D)** with 50% are shown. **(E–H)** As the same to above, images of disulfide cross-linked crystals immersed in precipitant-free solutions with different concentrations of DMSO.

The influence of an organic solvent on the stability of the cross-linked crystals were investigated by soaking them into the precipitant-free solutions containing different concentrations of DMSO (0−50% *v*/*v*) (Figure 5). DMSO is currently employed across the biomedical field due to its relatively low toxicity at dilute concentrations (Camp et al., 2020). Whereas the non-cross-linked crystals of SNAT-T4L were dissolved immediately in the precipitant-free solution without DMSO (Figure 5A), the cross-linked crystals maintained their appearance until in the presence of 40% (*v*/*v*) DMSO in the preservation solutions (Figures 5E–G). When the concentration of DMSO was 50% (*v*/*v*), they were destroyed but not dissolved, as seen in Figure 5H. The cross-linked crystals immersed into the solutions with 20% (*v*/*v*) DMSO diffracted to 1.50 Å resolution, but that immersed into the solution above 30% DMSO lost the diffraction ability. Interestingly, this loss in diffraction was recovered by back-soaking into the solution without DMSO, resulting in the collection of diffraction data at 1.10 Å resolution. This implies that the disorder of the crystalline phase at the concentration of DMSO is caused by the reversible unfolding (or partial unfolding) of proteins. Using ethanol or acetonitrile, commonly used for protein purification, their influence on the stability of the cross-linked crystals was also investigated. Consequently, in the presence of 30% (*v*/*v*) ethanol or 30% (*v*/*v*) acetonitrile, the cross-linked crystals preserved their shape and diffraction, as shown in Supplementary Table S1. Thus, the disulfide cross-linked crystals exhibited their improved stability against the osmotic stress and also, importantly, suggested their practical re-usability in actual drug screening tests.

**FIGURE 6:**
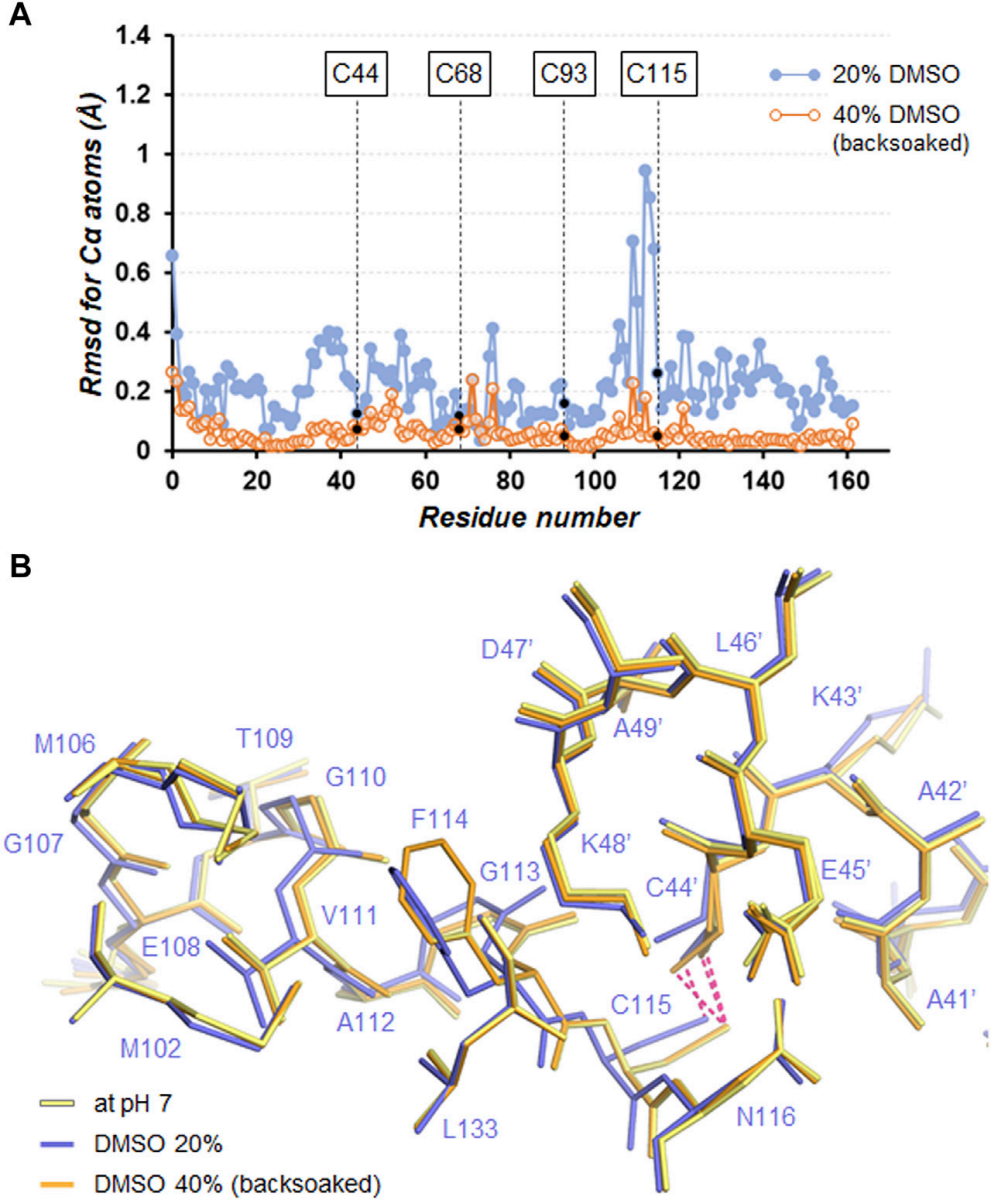
**(A)** Plot of the rmsd values for Cα atoms between the structures in the absence or presence of DMSO with respect to that at pH7. **(B)** Close-up view of the Cys115−Cys44’ cross-link site. Superimposed molecules are shown by line representation. The disulfide bonds are depicted by hotpink dashed lines.

Many studies have shed light on the effects of organic solvents on protein structure and function. In the presence of high concentration of organic solvents, intramolecular hydrogen bonds related to protein folding can be disrupted owing to their characteristics as hydrogen bond acceptors (Voets et al., 2010; Ghosh et al., 2014). Furthermore, the organic solvents can penetrate the protein interior and directly interact with buried hydrophobic residues, which leads to weakening the hydrophobic core structure (Bhattacharjya and Balaram, 1997; Dachuri et al., 2018). In a comparison of the SNAT-T4L structures with/without 20% (*v*/*v*) DMSO, little change in electrostatic interactions was observed, unlike when changing the pH (Table 3). A superimposition of the structures (rmsd of 0.26 Å for Cα atoms) indicated that the Cα positions between Met106 and Asn116 moved significantly as shown in Figure 6A. In the structure in the presence of 20% (*v*/*v*) DMSO, the side-chain of Phe114 shows a conformational change accompanied with partial cleavage of the Cys115−Cys44’ cross-link. Considering that these conformational changes were recovered in the crystal back-soaked into the preservation solution without DMSO, it suggests that the DMSO molecules act on the region between Met106 and Asn116. Actually, an electron density considered to be derived from DMSO was located near the Phe114 side-chain (Supplementary Figure S1A). In the active site filled with solvent molecules, some bulky densities were also observed, but it was difficult to assign the DMSO molecules with a single orientation (Supplementary Figure S1C). Such bulky electron densities were not confirmed in pH7 (only buffer) crystal (Supplementary Figures S1B, D). The Phe114 side-chain is involved in the formation of an internal hydrophobic core with the side-chains of Met102, Met106, Val111, and Leu133. The permeation of DMSO into the hydrophobic core may lose its core structure and initiate the protein unfolding leading to loss of the diffraction ability.

**TABLE 3:** Comparison of the electrostatic interactions observed in the structures in the absence or presence of DMSO. Selected interactions within 3.6 Å are listed, where their coordinate uncertainties were estimated from the Cruickshank’s DPI to be 0.06 Å for the structure soaked into a 20% (*v*/*v*) DMSO solution and 0.03 Å for that back-soaked into a DMSO-free solution, respectively.

Electrostatic interactions	DMSO 20%^a^	DMSO 40%^a^ (back-Soaked)	pH7
N-term (M1)−O^ε1^ (E64’)	2.92 (−0.17)	3.22 (+0.13)^b^	3.09
O^δ1^ (D10)−N^η2^ (R148)	2.80 (−0.01)	2.82 (+0.01)	2.81
O^ε2^ (E11)−N^η1^ (R145)	2.77 (+0.01)	2.75 (+0.01)	2.76
N^η2^ (R14)−O^ε2^ (E128’)	3.00 (+0.60)	3.10 (+0.70)^b^	2.40^b^
N^ζ^(K19)−O^δ2^ (D127’)	2.78 (−0.01)	2.78 (−0.01)	2.79
O^ε2^ (E22)−N^η1^ (R137)	2.89 (+0.05)	2.80 (−0.04)	2.84
N^δ1^(H31)−O^δ2^ (D70)	2.64 (+0.03)	2.63 (+0.02)	2.61
O^ε1^ (E45)−N^ζ^(K48)	2.92 (+0.11)^b^	2.82 (+0.01)^b^	2.81^b^
O^ε2^ (E45)−N^ζ^(K135’)	None	None	None
N^ε^(R52)−O^ε2^ (E62)	2.76 (−0.06)	2.82 (0)	2.82
N^ζ^(K60)−O^ε2^ (E64)	2.82 (−0.12)	3.18 (+0.24)	2.94^b^
O^δ1^ (D72)−N^η1^ (R76)	None	None	2.84
O^δ2^ (D72)−N^η1^ (R96’)	3.03	3.23^b^	None
N^η2^ (R76)−O^δ1^ (D89)	None	None	None
N^η2^ (R80)−O^ε1^ (E108)	3.00 (−0.51)^b^	2.80 (−0.71)^b^	3.51^b^
N^ζ^(K85)−O^δ2^ (D89)	2.94 (+0.25)	2.66 (−0.03)^b^	2.69
O^δ1^ (D92)−N^η1^ (R95)	2.88 (+0.02)	2.89 (−0.03)	2.86
O^δ1^ (D159)−N^ζ^(K162)	None	None	None

a Differences between the structures in the absence or presence of DMSO with respect to that at pH7 are in the respective parentheses.

b Either or both residues adopted alternative conformations predumably because the interaction was weakened.

## Conclusion

This study could provide one approach where possible residue pairs for intermolecular disulfide bridges can be selected based on the distance between the Cβ atoms. The disulfide cross-linked crystals of SNAT-T4L showed improved stability against soaking in acidic and basic solutions despite removing the precipitant component, but their diffraction ability disappeared under conditions where protein denaturation was induced. Furthermore, the crystals remained their diffraction ability even for soaking into a solution containing relatively high concentrations of 20% (*v*/*v*) DMSO. In the crystal structure, some DMSO molecules appeared to invade into the active site filled with solvent molecules, suggesting the accessibility of small compounds into the active site. Whether the disulfide cross-linked crystals can be put to practical use for drug screening, it would be necessary to perform soaking experiments using selected drug target proteins. In the case of induced-fit, mutation sites for cross-linking should be considered not to hamper the conformational changes.

## Data Availability

The datasets presented in this study can be found in online repositories. The names of the repository/repositories and accession number(s) can be found below: http://www.wwpdb.org/, 7XE5 http://www.wwpdb.org/, 7XE6 http://www.wwpdb.org/, 7XE7 http://www.wwpdb.org/, 7XE9 http://www.wwpdb.org/, 7XEA.
