# Supplementary material for: Creation of Cross-Linked Crystals With Intermolecular Disulfide Bonds Connecting Symmetry-Related Molecules Allows Retention of Tertiary Structure in Different Solvent Conditions
Source: Front Mol Biosci. 2022 Jun 8;9:908394. doi: 10.3389/fmolb.2022.908394 (PMC9213883; doi:10.3389/fmolb.2022.908394)
Supplement: Supplementary file 1 [file Table1.DOCX]

Supplementary Material

**TABLE S1.** Data collection statistics for the SNAT-T4L mutant in different buffer conditions. The statistics for the highest-resolution shell are presented in parentheses.

|  | **Precipitant solution** | **Distilled water** | **pH5** | **pH6** | **pH8** | **pH9** | **ethanol 30%** | **acetonitrile 30%** |
| --- | --- | --- | --- | --- | --- | --- | --- | --- |
| Beamline | NW12A at PF-AR | BL5A at PF | NW12A at PF-AR | NW12A at PF-AR | NW12A at PF-AR | NW12A at PF-AR | BL5A at PF | BL5A at PF |
| Cell constants (Å) | *a* = *b* = 59.91, *c* = 96.32 | *a* = *b* = 60.02, *c* = 96.49 | *a* = *b* = 60.12, *c* = 96.81 | *a* = *b* = 60.06, *c* = 96.91 | *a* = *b* = 60.02, *c* = 96.94 | *a* = *b* = 60.04, *c* = 97.09 | *a* = *b* = 59.99, *c* = 96.21 | *a* = *b* = 59.83, *c* = 96.31 |
| Resolution (Å) | 45.7 − 1.10 (1.14 − 1.10) | 45.8 − 1.05 (1.09 − 1.05) | 22.9 − 1.10 (1.14 − 1.10) | 22.0 − 1.05 (1.09 − 1.05) | 30.0 − 1.20 (1.24 − 1.20) | 30.0 − 1.15 (1.19 − 1.15) | 35.3 − 1.20 (1.24 − 1.20) | 22.8 − 1.25 (1.29 − 1.25) |
| Unique reflections | 81597 (8047) | 94140 (9291) | 82385 (8059) | 92735 (8852) | 63395 (5863) | 72137 (6892) | 63182 (6214) | 55622 (5324) |
| Redundancy | 10.4 (9.1) | 10.3 (9.3) | 10.2 (7.6) | 10.4 (8.1) | 10.1 (6.1) | 10.0 (5.6) | 10.5 (9.4) | 10.2 (7.6) |
| *I*/σ (*I*) | 20.5 (2.0) | 30.8 (5.2) | 23.7 (2.4) | 24.9 (2.0) | 25.9 (2.3) | 29.6 (3.1) | 25.7 (2.9) | 26.1 (2.4) |
| *R*_meas_^a^ | 0.077 (0.809) | 0.056 (0.359) | 0.083 (0.791) | 0.073 (0.884) | 0.070 (0.601) | 0.072 (0.485) | 0.064 (0.861) | 0.041 (0.684) |
| Completeness (%) | 99.9 (99.6) | 99.9 (99.9) | 99.5 (98.0) | 97.8 (94.4) | 99.3 (93.4) | 99.4 (96.4) | 100.0 (99.6) | 99.6 (97.1) |
| *CC*_1/2_^b^ | 0.999 (0.827) | 0.999 (0.968) | 0.998 (0.832) | 0.999 (0.780) | 0.999 (0.851) | 0.998 (0.899) | 0.999 (0.878) | 1.000 (0.846) |
| Wilson *B* (Å^2^) | 10.28 | 9.56 | 10.42 | 10.07 | 11.90 | 11.69 | 11.13 | 14.28 |

*^a^ R_meas_ is the multiplicity-weighted R_merge_. R_merge_ = Σ | I_hkl_ - <I_hkl_> | / Σ I_hkl_, <I_hkl_> is the mean value of I_hkl_.*

*^b^ CC_1/2_ values were calculated by splitting the data randomly in half.*
