# Supplementary material for: Creation of Cross-Linked Crystals With Intermolecular Disulfide Bonds Connecting Symmetry-Related Molecules Allows Retention of Tertiary Structure in Different Solvent Conditions
Source: Front Mol Biosci. 2022 Jun 8;9:908394. doi: 10.3389/fmolb.2022.908394 (PMC9213883; doi:10.3389/fmolb.2022.908394)
Supplement: Supplementary file 2 [file Image1.pdf]

Supplementary Material

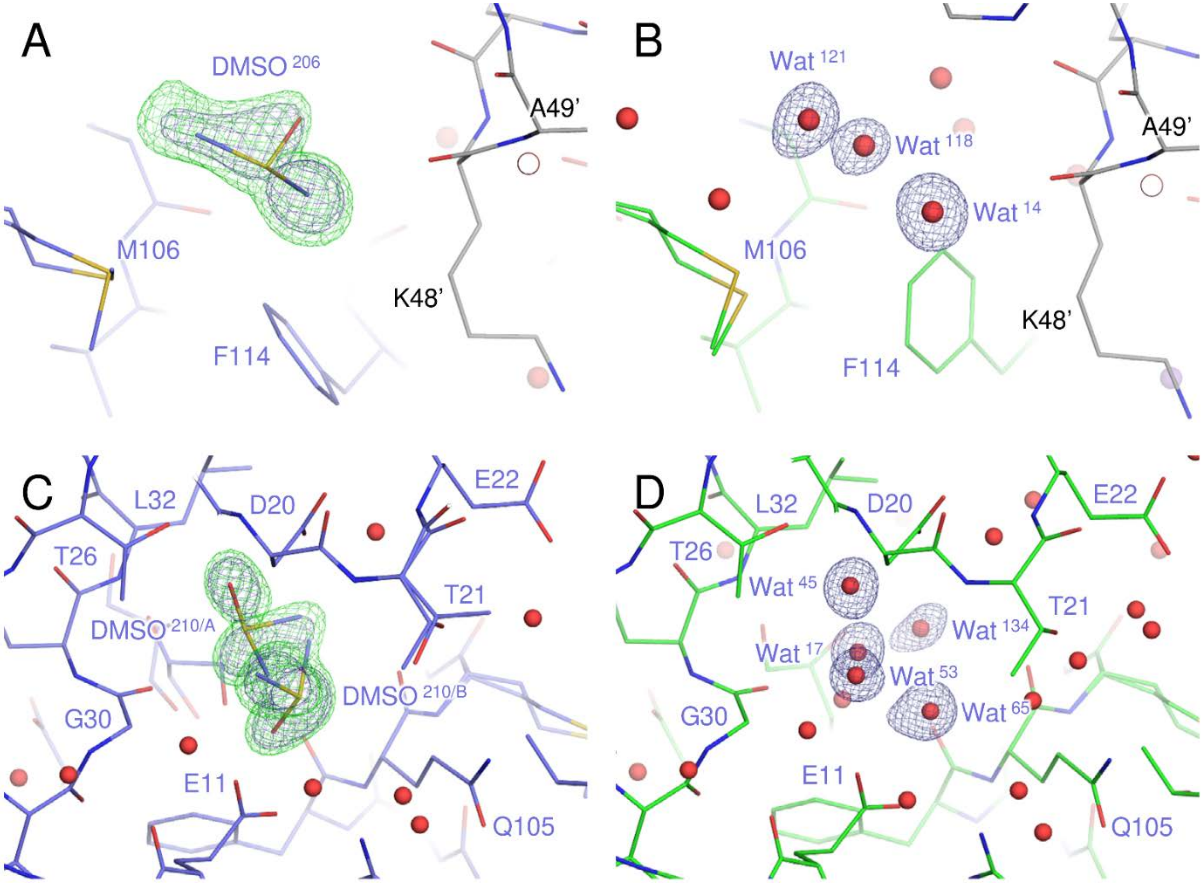

FIGURE S1. Electron densities of DMSO molecules in 20% DMSO soaked crystal. **(A)** DMSO molecule situated nearby the side chain of Phe114 and **(B)** the same position in pH7 (buffer) crystal. **(C)** DMSO molecules situated nearby the active site and **(D)** the same position in pH7 (buffer) crystal. Green meshes contoured at 3.2σ indicate polder maps (Liebschner et al., (2017). *Acta Cryst.* D73, 148−157), which are omit maps excluded the bulk solvent around the omitted region (DMSO molecules). Deep blue meshes contoured at 1.2σ indicate $2F_o - F_c$ electron-density maps.
